# An outbreak of *Salmonella *Enteritidis phage type 34a infection associated with a Chinese restaurant in Suffolk, United Kingdom

**DOI:** 10.1186/1471-2458-4-40

**Published:** 2004-09-01

**Authors:** Padmanabhan Badrinath, Torbjorn Sundkvist, Hamid Mahgoub, Richard Kent

**Affiliations:** 1 Southend Primary Care Trust & University of Cambridge, Harcourt Avenue, Southend on Sea, Essex, SS2 6HE, United Kingdom; 2 Suffolk Health Protection Unit, PO Box 170, South Building, St.Clements, Foxhall Road, Ipswich IP3 8LS, United Kingdom; 3 Department of Microbiology, Ipswich Hospital NHS Trust, Heath Road, Ipswich, Suffolk IP4 5PD, United Kingdom

## Abstract

**Background:**

On 30^th ^July 2002, the Suffolk Communicable Disease Control Team received notifications of gastrointestinal illness due to *Salmonella *Enteritidis in subjects who had eaten food from a Chinese restaurant on 27^th ^July. An Outbreak Control Team was formed resulting in extensive epidemiological, microbiological and environmental investigations.

**Methods:**

Attempts were made to contact everybody who ate food from the restaurant on 27^th ^July and a standard case definition was adopted. Using a pre-designed proforma information was gathered from both sick and well subjects. Food specific attack rates were calculated and two-tailed Fisher's exact test was used to test the difference between type of food consumed and the health status. Using a retrospective cohort design univariate Relative Risks and 95% Confidence Intervals were calculated for specific food items.

**Results:**

Data was gathered on 52 people of whom 38 developed gastrointestinal symptoms; 16 male and 22 female. The mean age was 27 years. The mean incubation period was 30 hours with a range of 6 to 90 hours. Food attack rates were significantly higher for egg, special and chicken fried rice. Relative risk and the Confidence interval for these food items were 1.97 (1.11–3.48), 1.56 (1.23–1.97) and 1.48 (1.20–1.83) respectively. Interviews with the chef revealed that many eggs were used in the preparation of egg-fried rice, which was left at room temperature for seven hours and was used in the preparation of the other two rice dishes. Of the 31 submitted stool specimens 28 tested positive for *S *Enteritidis phage type 34a and one for *S *Enteritidis phage type 4.

**Conclusion:**

In the absence of left over food available for microbiological examination, epidemiological investigation strongly suggested the eggs used in the preparation of the egg-fried rice as the vehicle for this outbreak. This investigation highlights the importance of safe practices in cooking and handling of eggs in restaurants.

## Background

Infection due to Salmonella is a major public health problem in England and Wales with reports of over 14,400 infections due to Salmonella in the year 2003 [1]. The most common serotypes responsible for human infection are *S *Enteritidis, *S *Typhimurium and *S *Virchow [2].

Although *Salmonella *enterica serovar Enteritidis phage types 4, 21 and 6 have been reported in previous outbreaks [3], phage type 34a is rare and reports of outbreaks due to this serotype are scarce in the literature. Apart from one report from Wales [4], we are not aware of any other outbreaks due to *S *Enteritidis PT 34a infection reported in the literature from United Kingdom. In this report, we present the results of an epidemiological investigation of an outbreak due to this rare phage type associated with a Chinese restaurant in Suffolk, United Kingdom.

## Methods

On 30^th ^July 2002, the Suffolk Communicable Disease Control team (SCDC) was informed by the consultant microbiologist that *S*. Enteritidis had been isolated from stool samples of five patients. All had recently eaten a meal in a local Chinese restaurant. Further enquires revealed that there were more patients with similar food history and gastrointestinal symptoms. An Outbreak Control Team was convened on 31^st ^July and it was decided that a full investigation should be carried out to identify the extent of the outbreak, the probable vehicle of infection and to advise on the appropriate control measures.

### Epidemiological

The environmental health department (EHD) staff initially gathered information from people who had become ill on a standard data collection form. In the initial stages of the investigation, it became apparent that all those who became ill had eaten or had bought a take away at the restaurant on 27^th ^July 2002. The information collected included name, address, sex, their symptoms and date of onset. The restaurant provided the list of food items that were served/sold on the day in question. This menu extended to 40 food items. This list was shown to the restaurant patrons and they were asked to state the food items they had eaten. A variety of ways was used to identify further cases including the technique of snowball sampling. This involved asking the patrons whether they were aware of any others who had similar symptoms and had eaten in the restaurant. General Practitioners providing primary care in the area were contacted and were requested to check for patients with gastrointestinal symptoms. The presenting symptoms of the patrons were diarrhoea, headache, abdominal pain and fever.

The next step involved interviewing all those who had eaten/bought food on 27^th ^July whether they became ill or not. The restaurant provided table-booking details. The following case definition was adopted for the outbreak. "Symptoms of acute gastroenteritis including one of the following: diarrhoea, vomiting or abdominal pain up to 96 hrs after having had a meal from the said restaurant including takeaway between 22 and 30 July 2002 and/or individuals who have positive stool sample for *S*. Enteritidis up to 96 hrs after having a meal from the restaurant including a takeaway between 22 and 30 July 2002".

An analytical investigation was carried out using a retrospective cohort design. Efforts were made to identify anyone who ate or bought food at the restaurant on 27^th ^July. Eligibility for membership of the cohort was defined as a person having the opportunity to eat any of the food items available on the day.

### Statistical methods

Data was entered in to Statistical Package of Social Sciences version 10 [5]. Food specific attack rates and the corresponding two tailed p vales were derived by Fisher's exact test [6]. Univariate relative risk (RR) and 95% Confidence Intervals (CI) were calculated using standard cohort analysis [7].

### Microbiological

Stool samples were requested from all who had eaten food from the restaurant on 27^th ^of July. Environmental sampling was not carried out as this was considered to be of limited value. There was no food left over from 27^th ^July, but three food samples were taken on 30^th ^July and sent for analysis to the food laboratory at Chelmsford Public Health Laboratory. Stool specimens were sent to Ipswich Hospital microbiology laboratory and were cultured for the presence of *Salmonella *sp. Isolates of Salmonella were forwarded to the Laboratory of Enteric Pathogens at Central Public Health Laboratory, Colindale for phage typing. Standard procedures were adopted for phage typing at the laboratory [8].

### Environmental

The EHD staff inspected the premises including verifying the procedures for hazard analysis and critical control point (HACCP). Egg storage and preparation of egg items were also investigated during the visit. Efforts were made to trace the egg trail back to the supplier.

## Results

### Epidemiological

Data were gathered from 52 subjects who had eaten food from the restaurant on 27^th ^July of whom 38 developed symptoms and 14 were free of symptoms. Of the 38 who became ill 16 were male and 22 were female. The mean age was 27 years. The mean incubation period was 30 hours with a range of 6 to 90 hours suggesting a point source outbreak. Two patients received hospital care and there were no deaths. No gastrointestinal illness was reported among the kitchen staff of the restaurant in the weeks before or during the outbreak.

On investigation of food preparation practices at the restaurant, it appeared dishes containing egg were the most likely vehicle for this outbreak. However, this information was not discussed when gathering data from the subjects. Data was gathered in a standardised format from all subjects to avoid any interviewer or recall bias.

We had a strong "a priori" hypothesis that people who had eaten egg or food that had come in to contact with egg were at an increased risk even before looking at the data and these were analysed first. During analysis it became apparent that illness was significantly associated with the three food items that contained egg or the egg rice mixture as shown by the food specific attack rates (Table 1) and the increased relative risks (Table 2). When many other food items eaten on the day including pork-fried rice and a variety of fish dishes were analysed none of which showed an increased attack rate or was significant in the cohort analysis.

**Table 1:** Specific attack rates of suspected foods

Food item	Eaten			Not eaten			p* value
		
	Ill	Not Ill	Attack rate (%)	Ill	Not Ill	Attack rate (%)	
Egg fried rice	31	5	86.1	7	9	43.8	0.002
Special fried rice	13	0	100.0	25	14	64.0	0.009
Chicken fried rice	9	0	100.0	29	14	67.4	0.04

* Fisher exact test

**Table 2:** Relative risk and 95% Confidence Intervals of suspected foods

Food Item	Relative Risk	95% Confidence Interval
Egg fried rice	1.97	1.11 – 3.48
Special fried rice	1.56	1.23 – 1.97
Chicken fried rice	1.48	1.20 – 1.83

### Microbiological

A total of 31 stool specimens were submitted to the laboratory from which *S*. Enteritidis was isolated in 29. Twenty-eight of these 29 isolates were confirmed to be PT 34a and one as PT4. No pathogens were isolated from food samples taken from the restaurant on 30^th ^July.

### Environmental

During the visit to the restaurant, the EHD staff reported that there was no evidence of hazard analysis and noted many cleaning and maintenance issues. Hand wash facilities were inadequate. The chef explained that after preparing the egg rice mixture, it was left out at room temperature for the rest of the evening, and reheated when ordered. This mixture was used for some of the other fried rice items. It was estimated that on the evening of the 27^th ^of July the egg rice mixture was left at room temperature for seven hours. The restaurant received eggs from a supplier in London every week and they were not refrigerated. Attempts to trace the egg trail were not successful.

### Control measures

The restaurant closed voluntarily on 31^st ^July and the EHD staff reassessed the situation on the evening of 1^st ^of August. As they were satisfied with the arrangements, the restaurant was allowed to reopen. The owner and restaurant staff were provided with information on proper cooking methods and the importance of undertaking HACCP.

## Discussion

The epidemiological investigation showed that eggs used in the preparation of egg-fried rice, which in turn was used in the preparation of some of the other rice items was the vehicle of infection. Isolation of the unusual phage type 34a strengthened the conclusion that eating from the restaurant was linked to a point source outbreak. In general, by the time investigations are initiated often no food material is available for laboratory analysis and the investigator has to rely on epidemiological evidence. The first step in identifying the source of an outbreak is the calculation of attack rates and the responsible food should have a significantly higher attack rate [9]. In this study, three types of food were found to have higher attack rates and were considered responsible for the outbreak (Table 1). All these items contained egg or egg rice mixture, which was left at room temperature for a long time. Cohort analysis also showed elevated RRs which were significant (Table 2). Seven sick patrons did not eat egg-fried rice. Descriptive analysis showed that all except one gave a history of eating chicken and or special fried rice.

Statistical methods have better power while there is an "a priori" hypothesis as shown in the study of summer excess of leukaemia [10]. We had an "a priori" hypothesis that food items containing eggs increased the risk of illness.

To our knowledge, this is the third report of an outbreak due to phage type 34a and the first of its kind in England published in the literature. In the UK, this phage type has been associated with travel abroad especially to southern Spain [11] and indigenous infections are rare. The restaurant received eggs from two sources and one of which was a packaging firm. Hence, it was not possible to determine the origin of the eggs. An outbreak due to closely related phage type 34, associated with an egg-containing dish in a Mexican restaurant in the United States has also been described [12]. There have been earlier reports of *S*. Enteritidis outbreaks associated with Chinese food businesses in England [13], Scotland [14] and the United States [15] although it is not clear whether any shortfalls in specific food handling techniques are responsible. In one instance [15], egg roll batter was made from pooled shelled eggs which were left at room temperature throughout the day.

The proportion of eggs infected with *S*. Enteritidis has been reported to be low [16] and hence the risk of acquiring infection from consuming a single raw egg is much lower. However, the practice of pooling shelled eggs together with storage at room temperature as happened in our outbreak promotes bacterial multiplication and a single contaminated egg can contaminate different types of food. The role of *S*. Enteritidis in causing food borne outbreaks is well known as it has the ability to contaminate eggs without causing discernible illness in the birds affected [17]. Eggs have been implicated as the source of Salmonella infection in many previous outbreaks [18-22]. Hayes *et al *[23] in their case control study in Wales found that undercooked hens eggs are an important risk factor for sporadic Salmonella infections.

We could not find any veterinary data on phage type 34 in British flocks. We searched the literature to determine whether there is any molecular relationship between phage type 34a and phage type 4. Hudson et al [24] based on the results of pulsed-field gel electrophoresis concluded that different *S*. Enteritidis phage types appear to be genetically related or clonal. Discussion on stability of phage types of S. Enteritidis can also be found in the literature. Conversion of phage type 4 to 24, phage type 23 to 8 and 4 to 7 have been reported. We could not find reports linking phage type 34a and phage type 4. In a recent public health investigation [25] of *S*. Enteritidis in raw eggshells, various serotypes of Salmonella were isolated from 23 out of 449 (5.1%) pooled samples labelled as originating from Spain. These sero/phage types included S. Enteritidis PT6a, PT5c, 13a, 14b, 58, PT6d, PT1, PT1c and PT12.

A few limitations of this study are to be noted. The origin of the suspected contaminated eggs could not be traced. Although trace back exercises are key in epidemic investigations, often they are not successful due to logistic and practical reasons. In our outbreak one of the suppliers to the restaurant turned out to be a packaging firm. During our investigation, we found that there were problems with the distribution system, which prevented us from pin pointing the origin of the contaminated eggs. A possibility always exists that we missed a few subjects from this investigation and not all could be persuaded to provide a stool sample. There was no single list of all the patrons who ate/purchased food on the evening. However, all efforts were made to contact the patrons and the outbreak caused considerable publicity in the local media. Hence, we are confident that we have included most patrons. Although the precise number of patrons who were not included will never be known we are confident that their number is small and might be in the region of 10 to 15.

Palmer [26] has pointed out the need to undertake outbreak investigations rapidly but at the same time with sound methodology. We tried to adopt the standard approach to investigating an outbreak including a retrospective cohort study. However, we did not attempt multivariate analysis due to the small number of subjects involved in the investigation.

*S*. Enteritidis PT4 was isolated from one of the subjects. Further investigation revealed that this subject had recently returned from holiday in continental Europe and had suffered mild symptoms before the meal.

In response to this and other outbreaks associated with eggs, a Public Health Investigation was launched in October 2002 in the UK to determine the rate of Salmonella contamination in eggs. Tests of nearly 4000 eggs showed that Salmonella was recovered from 5.3% of pooled eggs [25]. The Food Standards Agency has also produced a leaflet titled "Eggs – what caterers need to know" [27] which emphasises the importance of thoroughly cooking the eggs, buying eggs from reputable suppliers and use of pasteurised eggs when serving a vulnerable individuals.

## Conclusions

Investigation of this outbreak was greatly facilitated by the close cooperation between local EHD, Communicable Disease Control Team, microbiological laboratory and local health care providers. Although food samples from this point source outbreak were not available for microbiological culture, epidemiological evidence pointed to eggs containing dishes as the most likely source of the outbreak. This outbreak highlights the continuing hazards of raw eggs. It is likely that the use of pasteurised eggs and the adoption of safe food preparation practices would have prevented this outbreak.

## Competing interests

The first author (PB) served as the expert witness for the prosecution during the court proceedings.

## Authors' contributions

PB and TS conceived and designed the study and drafted the manuscript.

PB analysed the data

RK oversaw the microbiological investigation.

PB, TS, RK and HM all interpreted the results of the analysis and critically reviewed the manuscript.

All authors read and approved the final manuscript.

## Pre-publication history

The pre-publication history for this paper can be accessed here:



## References

[B1] CDR Weekly, Health Protection Agency. http://www.hpa.org.uk/cdr/PDFfiles/2003/cdr3303.pdf.

[B2] CDR Weekly, Health Protection Agency. http://www.hpa.org.uk/cdr/PDFfiles/2003/cdr2403.pdf.

[B3] CDR Weekly, Health Protection Agency. http://www.hpa.org.uk/cdr/PDFfiles/2002/cdr4502.pdf.

[B4] Linnane E, Roberts RJ, Mannion PT (2002). An outbreak of Salmonella enteritidis phage type 34a infection in primary school children: the use of visual aids and food preferences to overcome recall bias in a case control study. Epidemiol Infect.

[B5] Statistical Package for Social Sciences version 10,. http://www.spss.com.

[B6] Simple Interactive Statistical Analysis (SISA). http://home.clara.net/sisa/fisher.htm.

[B7] DJR Hutchon. Calculator for Confidence interval for Relative Risk. http://www.hutchon.freeserve.co.uk/ConfidRR.htm.

[B8] Hickman-Brenner FW, Stubbs AD, Farmer JJ (1991). Phage typing of Salmonella enteritidis in the United States. J Clin Microbiol.

[B9] Tansel O, Ekuklu G, Otkun M, Otkun MT, Akata F, Tugrul M (2003). A food-borne outbreak caused by Salmonella enteritidis. Yonsei Med J.

[B10] Badrinath P, Day NE, Stockton D (1997). Seasonality in the diagnosis of acute lymphocytic leukaemia. Br J Cancer.

[B11] Anonymous (1995). Salmonella enteritidis phage type 34 and holidays abroad. Commun Dis Rep Wkly.

[B12] McNeil MM, Sweat LB, Carter SL, Watson CB, Holloway JT, Manning R, Altekruse SF, Blake PA (1999). A Mexican restaurant-associated outbreak of Salmonella Enteritidis type 34 infections traced to a contaminated egg farm. Epidemiol Infect.

[B13] CDR weekly. Salmonella Enteritidis PT56 in Durham – final report. http://www.hpa.org.uk/cdr/archive04/news/news0504.htm#pt56.

[B14] Cowden J, Hamlet N, Locking M, Allardice G (2003). A national outbreak of infection with Salmonella enteritidis phage types 5c and 6a associated with Chinese food businesses in Scotland, summer 2000. Epidemiol Infect.

[B15] Boyce TG, Koo D, Swerdlow DL, Gomez TM, Serrano B, Nickey LN, Hickman-Brenner FW, Malcolm GB, Griffin PM (1996). Recurrent outbreaks of Salmonella Enteritidis infections in a Texas restaurant: phage type 4 arrives in the United States. Epidemiol Infect.

[B16] Centre for Disease Control and Prevention (1992). Outbreak of Salmonella enteritidis infection associated with consumption of raw shell eggs, 1991. MMWR.

[B17] Guard-Petter J (2001). The chicken, the egg and Salmonella enteritidis. Environ Microbiol.

[B18] Ejidokun OO, Killalea D, Cooper M, Holmyard S, Cross A, Kemp C (2000). Four linked outbreaks of Salmonella enteritidis phage type 4 infection – the continuing egg threat. Commun Dis Public Health.

[B19] Trepka MJ, Archer JR, Altekruse SF, Proctor ME, Davis JP (1999). An increase in sporadic and outbreak-associated Salmonella enteritidis infections in Wisconsin: the role of eggs. J Infect Dis.

[B20] Rejnmark L, Stoustrup O, Christensen I, Hansen A (1997). Impact of infecting dose on severity of disease in an outbreak of food-borne Salmonella enteritidis. Scand J Infect Dis.

[B21] Centre for Disease Control and Prevention (2003). Outbreaks of Salmonella serotype enteritidis infection associated with eating shell eggs – United States, 1999–2001. MMWR Morb Mortal Wkly Rep.

[B22] Irwin DJ, Rao M, Barham DW, Pencheon DC, Lofts C, Jones PH, O'Mahony M, Soltanpoor N, Ward LR, Threlfall EJ (1993). An outbreak of infection with Salmonella enteritidis phage type 4 associated with the use of raw shell eggs. Commun Dis Rep CDR Rev.

[B23] Hayes S, Nylen G, Smith R, Salmon RL, Palmer SR (1999). Undercooked hens eggs remain a risk factor for sporadic Salmonella enteritidis infection. Commun Dis Public Health.

[B24] Hudson CR, Garcia M, Gast RK, Maurer JJ (2001). Determination of close genetic relatedness of the major Salmonella enteritidis phage types by pulsed-field gel electrophoresis and DNA sequence analysis of several Salmonella virulence genes. Avian Dis.

[B25] CDR weekly. Public Health Investigation of Salmonella Enteritidis in raw shell eggs. http://www.hpa.org.uk/cdr/PDFfiles/2003/cdr0203.pdf.

[B26] Palmer SR (1995). Outbreak investigation: the need for 'quick and clean' epidemiology. Int J Epidemiol.

[B27] Food Standards Agency, Eggs-What caterers need to know. http://www.food.gov.uk/multimedia/pdfs/eggleaflet.pdf.

